# Spirituality, loneliness and mental health in Blackfeet American Indian adults

**DOI:** 10.1371/journal.pone.0325931

**Published:** 2025-06-13

**Authors:** Betty Henderson-Matthews, Reece Kothe, Skye Gilham, Zachary J. Wood, George Heavy Runner, Lester Johnson III, Mary Ellen Lafromboise, Melveena Malatare, Emily Salois, Neha A. John-Henderson

**Affiliations:** 1 Blackfeet Community College, Browning, Montana, United States of America; 2 Montana State University, Department of Psychology, Bozeman, Montana, United States of America; 3 Montana State University, Center for American Indian and Rural Health Equity, Bozeman, Montana, United States of America; University of Alberta, CANADA

## Abstract

Prior work documents a relationship between spirituality and mental health in American Indians. Separately, a robust literature links loneliness to indices of mental health. The current study is grounded in Community Based Participatory Research methods and investigates the relationship between spirituality, loneliness and indices of mental health. In a sample of 276 Blackfeet American Indian adults, a linear regression controlling for age, gender, education and marital status showed that higher levels of spirituality predicted lower levels of loneliness (β = −.31, t(266)=−5.34, p < .001, r^2^ change = .10). and lower levels of symptoms of depression= (β = −.24, t(267)=−4.02, p < .001, r^2^ change = .06) and anxiety (β = −.33, t(267)=−5.94, p < .001, r^2^ change = .11). Further analyses showed that higher levels of spirituality were linked to fewer symptoms of depression in part through lower levels of loneliness indirect effect (standard error, SE)= −.42 (.11), 95% CI= [−.65,-.23]. Similarly, higher levels of spirituality were linked to fewer symptoms of anxiety in part through lower levels of loneliness (indirect effect (SE)=−.46 (.12), 95% CI= [=−.70, −.26]. The findings offer preliminary evidence to support the notion that spirituality may confer benefits for mental health in part by decreasing loneliness for Blackfeet American Indian adults. Future work should investigate the social and behavioral pathways through which spirituality is linked to loneliness and mental health in this community.

## Introduction

American Indian spirituality is sacredly interwoven with the natural world, with diverse spiritual traditions and ceremonies connected to nature [1]. The very framework of creation comes from the natural world in the form of non-human, spirit-like creators that share close traits with animals and the natural world [1,2].The relationship between the creators and living beings that are created is akin to a dynamic and fluid dance; very alive and continuous and not existing in the past but rather in the present moment [2]. This lively relationship lends itself to an American Indian worldview that interprets the world as a living entity whose natural components are linked by a greater consciousness that extends beyond the physical body [2]. Therefore, spirituality encompasses every aspect of American Indian life and culture in such a way that it is not a part of a separate religious institution [3], but rather a deeply valued characteristic of life that is found in every aspect of existence both at the individual level up to larger groups and organizations [4,5]. To this end, American Indian spiritual beliefs center around an ideology of interconnectedness in which everything in the natural world including both living and inanimate things contain meaning and wisdom for interpersonal growth and greater connection to other people [4]. It is this interrelation among all things that provides unity among American Indians linking them together through various rituals that transcend tribal affiliation and provides a meaningful foundation for life meaning and purpose [5].

The relationship between spirituality and health and well-being dates to the 19^th^ and 20^th^ centuries, during which religion and spirituality was often viewed as negative, contributing to hysteria [6,7]. A robust and growing literature documents the potential for spirituality to confer benefits for health and well-being [7–9]. Accumulating evidence suggests spirituality is an essential component of mental health and wellness exhibited across various populations and serves as a protective factor for depression [10], and other mental health related illnesses. To this end, the research literature suggests that spirituality is an important health factor across humans and not isolated to specific spiritual traditions and practices [10]. The importance of spirituality as a protective factor can even be observed within the brain in the form of greater cortical reserves and a thicker cortex, which may combat depression by limiting the cortical thinning that is often found in individuals with depression [11]. In a correlational study conducted by Miller and colleagues, spirituality related to a 90% reduction of major depression symptoms for high-risk individuals with a family history of depression [11].

It has been proposed that spirituality relates to distinct human virtues including altruism and gratefulness, which may contribute to spirituality’s positive relationship with mental health [6]. Spirituality may also protect mental health by reducing risk for substance use disorder [12], or by affecting other health behaviors including sleep [13]. Higher levels of spirituality have also been linked to biological factors that are related to positive mental health [14–16]. Spirituality is also posited to contribute to better health and well-being through fostering and improving social relationships, expanding social networks, and through provision of social support [17]. Importantly, these proposed mechanisms have not been investigated in American Indian adults.

American Indian spiritual views and customs were endangered and put at-risk as European colonization began to dominate the land and native people in the late 1400s, evolving into cultural genocide with mass displacement and aggressive assimilation programs in the early 1800s [18–21]. In addition to the massive loss of life, there was significant psychological trauma because the American Indian relational view of spirituality and the natural world differed notably from European views [22]. Europeans sought expansionism through domination and control of the natural world, and Indigenous people. American Indian territory and resources were exploited through individual ownership of the land enforced with fences and barriers, and large livestock grazing operations that disrupted natural ecosystems [22]. The expansion of new settlements, individual settler interests, and resource acquisition were favored over unity with the preexisting Indigenous population and natural world.

As a result of European colonization, acts of genocide, ethnic cleansing, dislocation, loss of land, and mandatory boarding schools for American Indian children, American Indians were forced to submit to the dominant Anglo-Saxon culture [18–23]. American Indian spirituality and its associated ceremonies and traditions were outlawed until 1978 when the American Indian Religious Freedom Act (AIRFA) was passed with the intent being to suppress the inherent right of American Indians to believe and practice their traditional religions [24]. Such forced assimilation over the last 400 years has left deep scars and contributed to historical loss and the waning of American Indian traditions and cultural practices, including deeply rooted spiritual traditions and beliefs [22,23]. Notably, the consequences of historical trauma and associated losses can be passed from one generation to the next leading to a “cross generational cycle of trauma” [25] and lifespan trauma [19] which is perpetuated by potential physiological genetic adaptations [25].

To better understand the consequences of historical trauma and loss, Whitbeck et al. established a scale to empirically measure the frequency of thoughts about historical losses [23]. Recent research documents relationships between thoughts about historical loss and negative health conditions [26–28]. In one study, more frequent thoughts about historical loss related to lower rates of physical activity potentially contributing to risk for poor health [29], and separately more thoughts about historical loss were related to greater risk for cardiovascular diseases in a sample of American Indian adults [27]. A related large body of work documents links between historical trauma, associated loss and mental health in American Indians [30]. Related to this body of work, there are a growing number of interventions which utilize American Indian culture and spirituality to alleviate the wounds and damage associated with historical loss and trauma [31,32].

While acknowledging the negative consequence of colonization for American Indians, it is also important to recognize the long history of American Indians fighting back against cultural oppression, working to preserve cultural ways and traditions, and exhibiting resilience, survival and endurance in the face of historical and ongoing discrimination and oppression. For example, American Indian activism surged during the 1960’s with the work of many organizations including the American Indian Movement (AIM) focusing on advocating for American Indian rights and sovereignty [33]. The term survivance, engendered by Gerald Vizenor, refers to this enduring spirit and perseverance of American Indian people that extends beyond the trauma and loss associated with colonization. Survivance reflects presence over absence and the commitment to protecting American Indian culture, traditions and knowledge. The omnipresent nature of survivance is undeniably evident in American Indian stories, history, and can be observed in a variety of contexts in American Indians today [34].

### Enduring health inequities and the role of spirituality

Currently, American Indian populations have the highest rate of health disparities in the country as evidenced by worse overall health, higher incidence of depression, greater rates of suicide and lower life expectancy [35,36].Colonization and the associated historical trauma and loss of the American Indian way of life and spiritual practices has undoubtedly had a negative effect on the health and well-being of American Indians in the United States to this day.

The extant body of work on spirituality and health suggests that spirituality could be protective in the context of health for American Indians. For example, greater connection to ethnic identity, culture, and nature were all found to be health-protective for American Indians [3,20,37,38]. Spirituality and religion are key to helping American Indians reestablish traditional culture and experience post-traumatic growth [20]. A connection has also been established between land-based healing and spirituality, mindfulness and culture [38]. In a qualitative study of American Indians with cancer, a significant connection was found between spirituality, cultural traditions, and well-being [28]. Cultural and spiritual connection were also found to be protective factors for suicide prevention for American Indians and were more effective than mainstream suicide prevention interventions [32]. Utilizing an index of spiritual orientation, prior work found that practice of cultural spirituality was linked to significantly lower rates of suicide [39]. American Indian spirituality is interconnected to the natural world and all aspects of the way of being, including ethnic identity and culture. In this manner, spirituality serves as one of the potentially greatest, overarching factors related to American Indian health and healing from historical loss.

While acknowledging the negative implications of historical loss and trauma for American Indian mental health, more work is needed to understand whether and how spirituality may confer health benefits for American Indians. In the current study, we investigated whether higher levels of American Indian spirituality relate to better mental health in a sample of Blackfeet adults residing in the Blackfeet Nation in Northwest Montana. As described in the writings of John Ewers [40], the Blackfoot Indians lived in a world characterized by uncertainty, having endured health pandemics and the possibility of attack by surrounding tribes. However, Ewers notes that the Blackfeet did not face these stressors alone. Instead, they were surrounded by supernatural powers that they could call upon for protection and which would help them through their own challenges. The idea that everything in the universe has a spirit, that can either help or harm an individual depending on their interactions with it, is an idea that continues to be embraced by the Blackfeet. Further, there is no separation between spirituality and health, as they are seamlessly intertwined, such that being healthy spiritually translates to good health overall.

We note that Blackfeet spirituality and related knowledge is sacred. As such, the discussion of the specifics of Blackfeet spirituality in the current paper is limited by the parameters which limit transfer of this sacred knowledge. However, based on the noted interconnections between spirituality and health for the Blackfeet people, we hypothesize that spirituality in Blackfeet American Indians may relate to better mental health (I.e., fewer symptoms of depression and anxiety) in part by contributing to reduced sense of loneliness or perceived social disconnection. In our previous work, we have found evidence that perceived social connectedness is a health protective factor for Blackfeet American Indians [41,42]. Spirituality and active engagement in activities related to spirituality may increase feelings of connectedness and in doing so may promote better mental health.

One index of perceived connectedness is loneliness, or the unpleasant emotional state that is characterized by a perceived lack of social connection. This perception is distinct from objective social isolation or lack of social ties and instead is a subjective assessment of one’s social connectedness. Loneliness is often related to depression, a mental health condition that significantly impacts daily function and which is characterized by a persistent state of sadness and diminished interest or pleasure in activities. While loneliness and depression can be related to one another and have shared symptoms including fatigue, sadness, and feelings of worthlessness, there are important distinctions. Loneliness fluctuates, and circumstances can affect experiences of loneliness. In contrast, depression, a psychiatric disorder can be defined as a persistent state of low mood and reduced interest or pleasure in activities.

While depression may be exacerbated by or linked to social experiences and relationships, loneliness is an experience that is exclusively informed by social circumstances. A bidirectional relationship between loneliness and depression is supported by empirical data, with the potential for loneliness to exacerbate depression and for depression to worsen one’s experience of loneliness [43,44]. Loneliness has also been distinguished from depression in the Flathead American Indian community in previous work. Specifically, it was noted that the experience and reporting of loneliness did not require individual pathology and was not merely another word for depression. Rather, loneliness was described as a unique cultural, social, and psychological construct relating to belonging, interdependence, reciprocity, and compassion [45]. While the Blackfeet experience of loneliness has not been studied with the same cultural ethnography lens, prior work in the Blackfeet community indicates that loneliness and depression have distinct relationships with a range of health-relevant outcomes [41]. The current work is the first to test whether spirituality is linked to better mental health in Blackfeet American adults in part through lower levels of loneliness.

## Methods

The current project is founded upon a long-standing partnership between a Psychology Professor at Montana State University and Blackfeet Community members and faculty and students at Blackfeet Community College. This research program extends on longstanding partnerships between Blackfeet Community College and Montana State researchers, and a research program dedicated to improving health and addressing health inequities in the Blackfeet community [46]. The current research program and partnership is based on Community Based Participatory Research principles which emphasize the importance of an equitable partnership between researchers and community members. As such, a community advisory board comprised of Blackfeet Community members reviewed and helped to select and design the research questions, study design, selected measures, and guide data interpretation. Based on many years of pilot studies and research investigations, the research team developed the current study, “*Aa Koo Moo Waap*,” which can be translated to People Coming Together in the Blackfeet language, Aamsskaapipikani. The overarching goal of A*a Koo Moo Waap* is to elucidate the relationship between social connectedness and health in the Blackfeet Community over a 2-year period. Data collection for the data presented in the current manuscript began on 04/14/2023 and ended on 04/22/2023. The study was approved by the Blackfeet Nation Institutional Review Board and all participants provided written informed consent before participation.

The research team includes one Psychology faculty member from Montana State University, graduate students from Montana State University, a Community Research Associate from Montana State University (a member of the Blackfeet Community), a project coordinator at Blackfeet Community College (a member of the Blackfeet Community), a faculty member from the Blackfeet Community College (a member of the Blackfeet Community), several students and interns from the Blackfeet Community College (all members of the Blackfeet Community, and a Community Advisory Board (comprised of four Blackfeet Community Members).

During the first year of the project, the research team met collaboratively to develop the study goals, study design, select appropriate measures, obtain IRB approval of necessary amendments to the protocol, and to begin recruitment. The research team recruited Blackfeet community members aged 18–65 for the project. Recruitment began in January of 2023 and finished in March of 2023. We planned to collect data from 280 Blackfeet Community Members. The sample size was based on minimum requirements for the data analysis plan for longitudinal models and to account for potential attrition. The research team used community connections for study advertising and tried to include community members from all the communities in the reservation.

Eligibility criteria included being between the age of 18–65, self-identification as American Indian, and current residence in the Blackfeet Nation. Eligibility was confirmed using a survey on the Qualtrics platform for most participants before data collection began. For other participants, who did not have access to email or the internet, we confirmed eligibility over the phone or using an in-person interview. Once eligibility was confirmed, participants were scheduled to come to the Blackfeet Community College for data collection. There were four participants who completed the screening that were not eligible for participation. Only two participants who were eligible did not show up for data collection.

Data collection took place in April of 2023. Participants were told at the time the appointment was made that they would need to fast for 12 hours prior to data collection, and to consume only water in the 12 hours prior to their appointment. We also sent out text and email reminders the day before their appointments to remind them to fast prior to coming to the college. Upon arriving at their scheduled appointment, the project coordinator went over all the procedures and the purpose of the study. After providing this information, community members who chose to participate in the study provided informed consent. Immediately after providing informed consent, Blackfeet Community College student interns and nursing students measured participants’ resting pulse rate, and systolic and diastolic blood pressure using an Omron blood pressure device and measured their waist circumference, height and weight. Participants moved to a separate room for blood draws. Participants provided blood samples to measure risk for cardiometabolic disease and inflammatory disease. These measures were collected as part of the larger Aa Koo Moo Waap project and will not be discussed here.

Finally, participants completed a survey using the Qualtrics platform on a desktop computer or using pen and paper if that was their preference. The survey included multiple measures of social connectedness, mental health, physical health, sleep health, trauma (recent and childhood), civic engagement, perceived discrimination and other psychosocial variables. The current manuscript is based on data from Wave 1 of the *Aa Koo Moo Waap* project. All participants were paid $100 for participation in this wave of data collection.

### Measures

#### Demographics.

Participants self-reported age, gender, highest level of education and marital status. These variables were used as covariates in our analyses of relationships between spirituality, loneliness and mental health.

### Spirituality

We used the Native American Spirituality Scale (NASS) to measure of levels of spirituality [47]. The scale was developed in collaboration with members of a Southwest tribe by modifying items used in the Daily Spiritual Experience Scale (DSES) [48]. The scale lists 12 experiences related to Native American Spirituality, and participants are asked to indicate how often they have had these experiences. Example items include, “I wake up early and pray to Creator/ancestors,” “I believe everything is alive with a spirit,” I participate in cultural/faith related activities.” The response options include *many times a day (5), every day (4), most days (3), some days (2), once in a while (1),* and *never or almost never (0).* In the current study, the NASS demonstrated good reliability (Cronbach alpha = . 95).

### Loneliness

The Short Loneliness Scale (LON) consists of 3 questions measuring perceived loneliness [49]. Using a 4-point scale, participants indicate the frequency with which they typically feel isolated, lacking in companionship or left out. In the current sample, the LON demonstrated good reliability (Cronbach alpha = .87).

### Symptoms of depression and anxiety

We used the Hospital Anxiety and Depression Scale as a measure of current symptoms of depression and anxiety [50]. The HADS scale has 14 items, 7 of which measure symptoms of depression, and 7 of which measure symptoms of anxiety. Participants respond to each item using a 4-point scale (0–3), with possible scores ranging from 0–21 for symptoms of depression, and 0–21 for symptoms of anxiety. Higher scores reflect more symptoms. Cronbach alpha in the current sample was 0.67 for the depression subscale and 0.83 for the anxiety subscale.

### Statistical analyses

Analyses were conducted using IBM SPSS Statistics (Version 29). All variables were converted to z-scores before use in statistical analyses. We used z-scores to allow us to compare effect sizes for variables using different scales. To test for main effects of spirituality on mental health and on loneliness, we used hierarchical linear regressions with the covariates of age, gender, highest level of education and marital status. To test for indirect effects of spirituality on mental health through loneliness, we used a bootstrapping approach where a point estimate of the indirect effect was derived from the mean of the 5000 estimates of the indirect pathways, and 95% confidence intervals (CIs) were computed using the cutoffs for the 2.5% highest and lowest scores of the distribution. Indirect effects were considered statistically significant when the CI did not include 0 [51].

## Results

Descriptive statistics and bivariate correlations are listed in Table 1. Age was positively related to highest level of education and anxiety symptoms, women had higher levels of education, spirituality was negatively related to loneliness and symptoms of anxiety and depression, and loneliness was positively related to symptoms of anxiety and depression.

**Table 1:** Descriptive statistics and bivariate correlations.

Variable	M	SD	Range	1.	2.	3.	4.	5.	6.	7.
1. Age	38.41	14.41	18-76	–	.080	.169**	−.088	−.032	−.197**	−.070
2. Gender	64.4% Female				–	.191**	.009	.111	.118	−.023
3. Education	47.3% High school diploma or less		1-6			–	.056	.106	−.077	−.178**
4. Native American Spirituality	28.49	17.29	0-67				–	.303**	−.307**	−.220**
5. Loneliness	5.84	2.40	3-12					–	.449**	.448**
6. Anxiety Symptoms	8.95	4.36	0-21						–	.653**
7. Depressive Symptoms	5.66	3.32	0-15							–

### Spirituality and mental health

We used a hierarchical linear regression to investigate the relationship between spirituality (as measured with the NASS) and symptoms of depression and anxiety (as measured with the HADS). In Step 1 of the hierarchical linear regression, we entered the covariates of age, gender, education, and marital status, and entered spirituality in Step 2. Spirituality was a statistically significant predictor of symptoms of depression and anxiety (β = −.24, t(267)=−4.02, p < .001, r^2^ change = .06), and (β = −.33, t(267)=−5.94,p < .001, r^2^ change = .11) respectively. See Table 2 and 3 for the results of these regression models.

**Table 2:** Summary of hierarchical linear regression model with spirituality predicting symptoms of depression.

Predictor	*B*	95% CI	β	*p*	R^2^
**Step 1**					
Age	−0.01	[-0.04, 0.02]	−0.03	0.64	
Gender	0.05	[-0.67,0.77]	0.10	0.10	
**Education**	**−0.41**	**[-0.70, 0.12]**	**−0.17**	**0.01**	
Marital Status	−0.37	[-1.25, 0.52]	−0.05	0.42	
					**0.02**
**Step 2**					
Age	−0.01	[-0.04, 0.02]	−0.05	0.45	
Gender	0.08	[-0.62, 0.79]	0.01	0.82	
**Education**	**−0.44**	**[-0.72, 0.15]**	**−0.18**	**<0.01**	
Marital Status	−0.43	[-1.29, 0.43]	−0.06	0.33	
**Spirituality**	**−0.05**	**[-0.07, -0.03]**	**−0.23**	**<0.001**	
					**0.08**

**Note: Gender was coded as follows: Female = 1, Male = 2, marital Status was coded as 0 = unmarried, 1 = married. Statistically significant predictors are listed in bold.**

**Table 3:** Summary of hierarchical linear regression model with spirituality predicting symptoms of anxiety.

*Predictor*	*B*	*95% CI*	*β*	*p*	*R* ^*2*^
***Step 1***					
***Age***	***−0.06***	***[-0.10, -0.02]***	***−0.19***	***<.0.01***	
***Gender***	***1.14***	***[0.20, 2.01]***	***0.15***	***0.02***	
*Education*	*−0.22*	*[-0.59, 0.16]*	*−0.07*	*0.25*	
*Marital Status*	*−0.14*	*[-1.29, 1.01]*	*−0.01*	*0.81*	
					***0.05***
***Step 2***					
***Age***	***−0.07***	***[-0.10, -0.03]***	***−0.22***	***<.0.001***	
***Gender***	***1.19***	***[0.31, 2.08]***	***0.15***	***0.01***	
*Education*	*−0.27*	*[-0.62, 0.09]*	*−0.09*	*0.14*	
*Marital Status*	*0.26*	*[-1.33, 0.83]*	*−0.03*	*0.64*	
***Spirituality***	***−0.08***	***[-0.11, -0.06]***	***−0.33***	***<0.001***	
					***0.16***

**Note: Gender was coded as follows: Female = 1, Male = 2, marital status was coded as 0 = unmarried, 1 = married. Statistically significant predictors are listed in bold**

### Spirituality and loneliness

We used a separate hierarchical linear regression predicting loneliness with the previously described covariates in Step 1, and spirituality in Step 2. Spirituality was a statistically significant predictor of loneliness (β = −.31, t(266)=−5.34,p < .001, r^2^ change = .10). The full results from this regression model are included in Table 4.

**Table 4:** Summary of hierarchical linear regression model with spirituality predicting loneliness.

Predictor	*B*	95% CI	β	*p*	R^2^
**Step 1**					
Age	−0.00	[-0.02, 0.02]	−0.02	0.80	
Gender	0.44	[−0.08,0.97	0.10	0.10	
**Education**	**0.01**	**[-0.21, 0.22]**	**0.00**	**0.06**	
**Marital Status**	**−0.59**	**[-1.23, 0.05]**	**−0.11**	**0.07**	
					**0.01**
**Step 2**					
Age	−0.01	[-0.03, 0.01]	−0.04	0.51	
**Gender**	**0.47**	**[-0.03, 0.97]**	**0.11**	**0.07**	
Education	−0.02	[-0.22, 0.19]	−0.01	0.88	
**Marital Status**	**−0.65**	**[-1.26, -0.04]**	**−.12**	**0.04**	
**Spirituality**	**−0.04**	**[-0.06, -0.03]**	**−0.31**	**<.001**	
					**0.11**

**Note: Gender was coded as follows: Female = 1, Male = 2, marital Status was coded as 0 = unmarried, 1 = married. Statistically significant predictors are listed in bold**

### Indirect effect of spirituality on mental health through loneliness

Using the previously described method [51], we found a statistically significant indirect effect of spirituality on symptoms of depression through loneliness (indirect effect (standard error, SE) = −.42 (.11), 95% CI= [−.65,-.23], and on symptoms of anxiety (indirect effect (SE)=−.46 (.12), 95% CI= [−.70,-.26].

## Discussion

To our knowledge, the current paper is the first to investigate whether spirituality is related to mental health outcomes through loneliness in a sample of American Indian adults. We hypothesized that these relationships would exist based on prior evidence of a relationship between spirituality and health in American Indians [52–54] and based on our own work indicating links between social connectedness and mental health in the Blackfeet community [41,42].

The findings reported here suggest that one pathway through which American Indian spirituality may be health-protective in the context of mental health is by reducing perceived social disconnection, or loneliness. In the current sample of Blackfeet American Indian adults, independent of covariates related to mental health including age, gender, education, and marital status, higher levels of American Indian spirituality, as measured by the Native American Spirituality scale, predicted lower levels of symptoms of both anxiety and depression, and lower levels of loneliness. The observed relationships between spirituality and symptoms of depression and the spirituality and symptoms of anxiety were mediated in part by levels of loneliness.

According to the American Indian worldview, a person does not exist as an independent entity but rather is viewed as an extension of their family, their community, their tribe, and more broadly, the universe [55]. The findings reported here, can be contextualized within this characterization of the American Indian worldview. Specifically, greater endorsement of American Indian spirituality and its associated values and beliefs may increase one’s sense of connectedness to others in their family, community and tribe, reducing feelings of loneliness, and ultimately may foster better mental health. These pathways may also be rooted in the American Indian conceptualization of health and well-being which does not distinguish between spiritual, physical, mental, and emotional domains, and instead emphasizes the interdependence between these domains [56]. Our findings are in line with this acknowledged interdependence, providing further evidence of an important relationship between spirituality and mental health through satisfaction with one’s social relationships (i.e., loneliness).

More broadly, the current findings add to a burgeoning literature on resilience factors specific to American Indian adults. In a recent scoping review on factors connected to health resilience in American Indian and Alaska Native health adults, social factors were the most common source of health resilience [57]. This review acknowledged that while social factors were the most frequent source of health resilience documented in the literature, the documented resilience domains of social, psychological, and spiritual factors likely interact and affect one another in complex ways. The current study provides support of this idea, with the observed relationships between spirituality, social connection and indices of mental health.

As previously discussed, the distinction between loneliness and depression and the bidirectional relationship between the two has been acknowledged across racial and ethnic groups and in the Flathead American Indian community [43–45]. The relationship between loneliness and depression or symptoms of depression in daily life is likely complex and nuanced. Future investigations could elucidate day-to-day relationships between these variables by measuring these constructs daily to understand the temporal relationship between these two factors as they unfold in daily life. Furthermore, qualitative measurement of the experience of loneliness in the Blackfeet community could contribute to a more developed understanding of what loneliness means to Blackfeet American Indian adults.

The current work does not investigate the pathways through which spirituality may promote connectedness. One possibility is that community members who are more spiritual may be more likely to participate in spiritual events and ceremonies and thus may have more opportunities to connect with other community members. It is also possible that because of the emphasis on interconnectedness in American Indian spirituality, those who are highly spiritual feel less isolated and disconnected.

The data from the current investigation is limited by its cross-sectional nature. Due to this limitation, it is not possible to infer causality. Thus, it is possible that lower levels of loneliness promote higher levels of spirituality, which then reduces symptoms of depression and anxiety. In future work, momentary assessments of loneliness, spirituality, and symptoms of depression and anxiety could shed light on directionality of these relationships. Since the *Aa-Koo-Moo-Waap* study is a longitudinal study, we will have the opportunity in subsequent waves of data collection to examine whether spirituality prospectively predicts changes in mental health and other relevant health outcomes. Overall, the findings here present initial evidence that higher levels of spirituality are linked to better mental health, in part through its association with lower levels of loneliness.

Our findings are in line with a growing call for interventions working to reduce American Indian health inequities to be grounded in indigenous world views [19,20,32,38]. American Indian spiritual beliefs are an essential aspect of American Indian culture and health. Therefore, continuing to expand and deepen research into the pathways and mechanisms through which spirituality is health protective for American Indians is imperative to addressing health inequities and acknowledging and healing the wounds of the past.
